# Construction of a Simple and Extremely Low-Cost 3D-Printed Ultrasound Model for Training in the Pericapsular-Nerve Group (PENG) Block Technique

**DOI:** 10.7759/cureus.111874

**Published:** 2026-07-01

**Authors:** Shane Moore

**Affiliations:** 1 Anaesthesia, Children's Health Ireland, Dublin, IRL

**Keywords:** ‎3d printing, pericapsular nerve group block (peng), phantom trainer, postoperative pain relief, regional anesthesiology

## Abstract

3D printing has multiple applications in the practice of medicine, including but not limited to the construction of 3D models to facilitate training in ultrasound-guided regional anaesthesia. Such models are typically far less expensive than commercially developed "phantom" trainers, making them particularly attractive in scenarios where budget constraints may impact regional anaesthesia training opportunities. The PEricapsular Nerve Group (PENG) block is a relatively recently described regional anaesthesia technique that is particularly useful in providing post-operative analgesia to patients undergoing hip replacement. To date, there are very few commercially available phantom models that permit the practice of the PENG block ex vivo, and those that do exist are considerably costly to purchase. This report describes the process of using readily accessible home 3D printing technology to create an extremely low-cost, simple-to-assemble phantom of the hip region, suitable for practicing the needling techniques necessary to carry out the PENG block. It may be of particular interest to regional anaesthesia training centres where funding issues present an obstacle to purchasing commercial models. The report demonstrates construction feasibility and preliminary sonographic usability of such a phantom, without formal evaluation of trainee learning, expert-rated fidelity, and comparative educational value. Free, open-source software and 3D models were used to prepare an appropriate pelvic model for printing. Stereolithography (SLA) 3D printing technology was used to produce a resin-based model of the pelvis, with gelatine utilised to create a tissue layer encompassing the model. A sonographic representation of the psoas tendon (a notable landmark in the block) was created, utilising pasta strands encompassed in nitrile and embedded in the gelatine at the expected location along the pelvic brim. The ultrasound characteristics of this phantom were reasonable, and the phantom permitted multiple needling attempts. The total cost for the production of this phantom was slightly over 12 euros (excluding initial 3D printer purchase and post-processing materials), and the total preparation time was 20 hours, including print time, print pre- and post-processing time, and gelatine setting times. This 3D phantom was produced using home 3D printing technology and affordable, readily available materials, for an exceptionally low cost and relatively fast production time. The limitations of the phantom include a short shelf life if not frozen, simplified anatomy, and relatively low ultrasonographic soft-tissue fidelity. Additional strategies for improving these aspects are discussed and could be implemented if desired.

## Introduction

3D printing is a technology that has multifaceted applications in the practice of medicine [1,2], not least of which is the construction of low-cost anatomical models to enable clinicians to practice various technical skills [3]. These skills include ultrasound-guided regional anaesthesia (USGRA) techniques [4,5], wherein clinicians aim to place a needle in close proximity to nerves or other anatomical landmarks in order to deliver local anaesthetic for analgesia or anaesthesia [6].

Such so-called "phantom" models offer trainees invaluable opportunities to practice the techniques and refine hand-eye coordination in a safe environment, without risk of patient complications [7].

Models for USGRA are commercially available but can be prohibitively expensive [8]. Compounding the issue is that the field of regional anaesthesia offers a wide variety of different techniques, many of which would require a different phantom to be purchased in order to facilitate effective practice.

3D printing of these models, using relatively low-cost technologies and materials, can help bridge this gap and offer ample opportunity for modifying and updating the models as required [9-11]. This may be particularly attractive to smaller anaesthetic departments or departments where the funding of regional anaesthesia training may be an issue.

The PEricapsular Nerve Group (PENG) block is a relatively new regional anaesthesia technique that offers pain relief to patients undergoing hip surgery, potentially without compromising their motor function [12]. The preservation of motor function enables the patient to participate in physical rehabilitation faster than those patients with a more traditional block - a desirable characteristic that may be associated with improved patient outcomes.

With the PENG block, an operator aims to inject local anaesthetic along the pubic ramus, where it is believed to contact and anaesthetise small articular nerve fibers arising from the femoral and accessory obturator nerves that innervate the anterior capsule of the hip [13]. In practice, this involves imaging the bony crest of the ilium with ultrasound, between the iliopubic eminence and the anterior inferior iliac spine. Thereafter, the operator guides the needle to strike bone lateral to the psoas tendon, before injecting local anaesthetic in this plane, superficial to the ilium and underneath the muscle of the iliacus, where said articular fibres are thought to lie [12].

The bony landmarks and endpoint of this block make it uniquely suited to 3D printing. Up until recently, no 3D-printed phantoms for the PENG block were reported, but in October 2025, Termos et al. published a letter describing their hybrid 3D-printed and meat PENG phantom [14]. At the date of publishing of this article, there appear to be few commercially available phantom trainers specific to the PENG block. Those found that do facilitate PENG training are currently sold for an excess of 2500 euros [15], making 3D printing a particularly attractive alternative.

The goal of this article was to produce a simple, low-cost 3D phantom using easily obtainable materials to simulate tissues, bones, and tendons in order to facilitate practice of image acquisition and needle guidance for the PENG block. While aiming to demonstrate the construction feasibility and preliminary sonographic usability of such a phantom, formal evaluation of trainee learning, expert-rated fidelity, and comparative educational value analysis was not undertaken at this juncture. Efforts were taken to streamline the process and make it as fast and economical as possible to produce. It represents a lower fidelity, but simpler and cheaper alternative to the work described by Termos et al. [14].

## Technical report

Construction of the 3D-printed pelvis

A free, open-source stereolithography (SLA) file of the pelvis was obtained, under creative commons 4.0 international license from an online STL website [16]. As the PENG block is frequently a unilateral block, the .stl file was modified in Meshmixer. This modification involved carrying out sequential plane cuts in order to leave a single hemipelvis with a minimum of vertebrae (Interactive Model 1). This facilitated the production of the model by reducing the material required for printing and enabled building in one single piece, which may otherwise be an issue for 3D printers with limited build plate capacity.

**Video 1:** Stereolithography (STL) file after modification This is the .stl model post-modification in Meshmixer. The original is available in Ref [16], under creative commons 4.0 international license from an online STL website. Using the "Plane Cut" function in Meshmixer, the original model was divided down the middle to create a hemipelvis, and the superfluous vertebrae were then removed using the same technique. Finally, the posterior aspect of the sacrum was removed using another plane cut to create a flat base, allowing the model to sit securely in the container.

**Figure 1:**
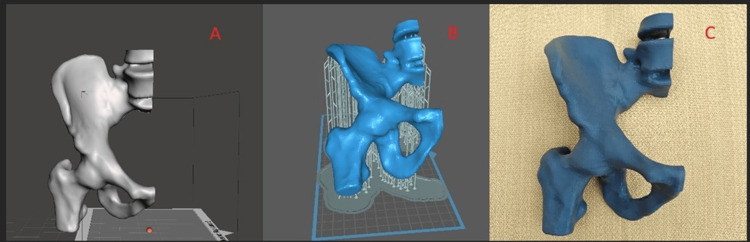
(A) Model modification in Meshmixer, (B) orientation and preprocessing in CHITUBOX, and (C) finished product

This modified .stl file (Figure 1A) was then prepared for 3D printing in Chitubox (a free slicer software utilised for the preparation of SLA 3D prints; CBD-Tech Co., Ltd., Shenzhen, China). Preparation involved hollowing of the model to a 3.0 mm wall thickness (again, to reduce 3D print material) with the insertion of six drainage holes on the bottom of the print (5.0 mm diameter) in order to facilitate liquid resin outflow.

The object was then inclined to 40 degrees and supported using Chitubox's automatic support settings - involving heavy supports at 50% density, using a skate raft for build plate adherence. Chitubox was then used to slice the image for 3D printing (Figure 1B).

The printer used was an Elegoo Saturn Ultra 3 SLA/resin printer (Elegoo, Shenzhen, China), with a 0.05 mm layer height and 2.5 second exposure time. Six bottom layers were printed with an exposure time of 35 seconds each to ensure build plate adherence. Lift speeds were set to 90 mm/min and retract speeds of 320 mm/min. The two-stage build plate lift distance was set to 3 + 4 mm, and the UV resin utilised was Elegoo Space Grey (Elegoo, Shenzhen, China).

The total build time for the 3D print was six hours and 18 minutes, and a total of 184 mL of resin was utilised.

Postprocessing was completed utilising a two-step isopropyl alcohol bath to remove excess liquid resin. This was followed by final curing using an ultraviolet lamp (three minutes per side - Navanino 40W Fast UV LED Nail Dryer - PZC-001 ) and the removal of print supports (Figure 1C). 

As is good practice with handling photosensitive liquid resin, personal protective equipment (PPE) was utilised at all stages of handling until postprocessing was completed. This included nitrile gloves, safety glasses, and a respirator. Processing and postprocessing took place in a well-ventilated area. Leftover resin supports were also washed and cured before being disposed of. Contaminated isopropyl alcohol can be reused for further washes as required, or left to sediment before being cured with ultraviolet light and recycled, with the sediment discarded. Table 1 presents the 3D print construction summary.

**Table 1:** 3D print construction summary

Printer model	Elegoo Saturn 3 Ultra
Resin type	Elegoo Space Grey
Layer height	0.05 mm
Exposure time	2.5 seconds
Bottom layers	6
Bottom layer exposure time	35 seconds
Wall thickness	3.0 millimetres
Drainage hole parameters	Number of holes = 6
	Diameter = 5 millimetres
Orientation to build a plate	+40 degrees
Chitubox support settings	Heavy (automatic)
	50% density
	45-degree angle
Chitubox raft settings	Shape = Skate
	Slope = 30 degrees
	Thickness = 1.5 millimetres
Total resin volume	184 millilitres
Total print time	6 hours, 18 minutes

Creating a replica of soft tissue

Soft tissue was simulated by adding two separate layers of gelatine over the model, which had been placed in a plastic container (35x21x12 cm; D-Clutter Transparent Storage Box & Lid; RSW International Limited, Greater Manchester, UK) and lined with plastic film to facilitate easy removal.

**Figure 2:**
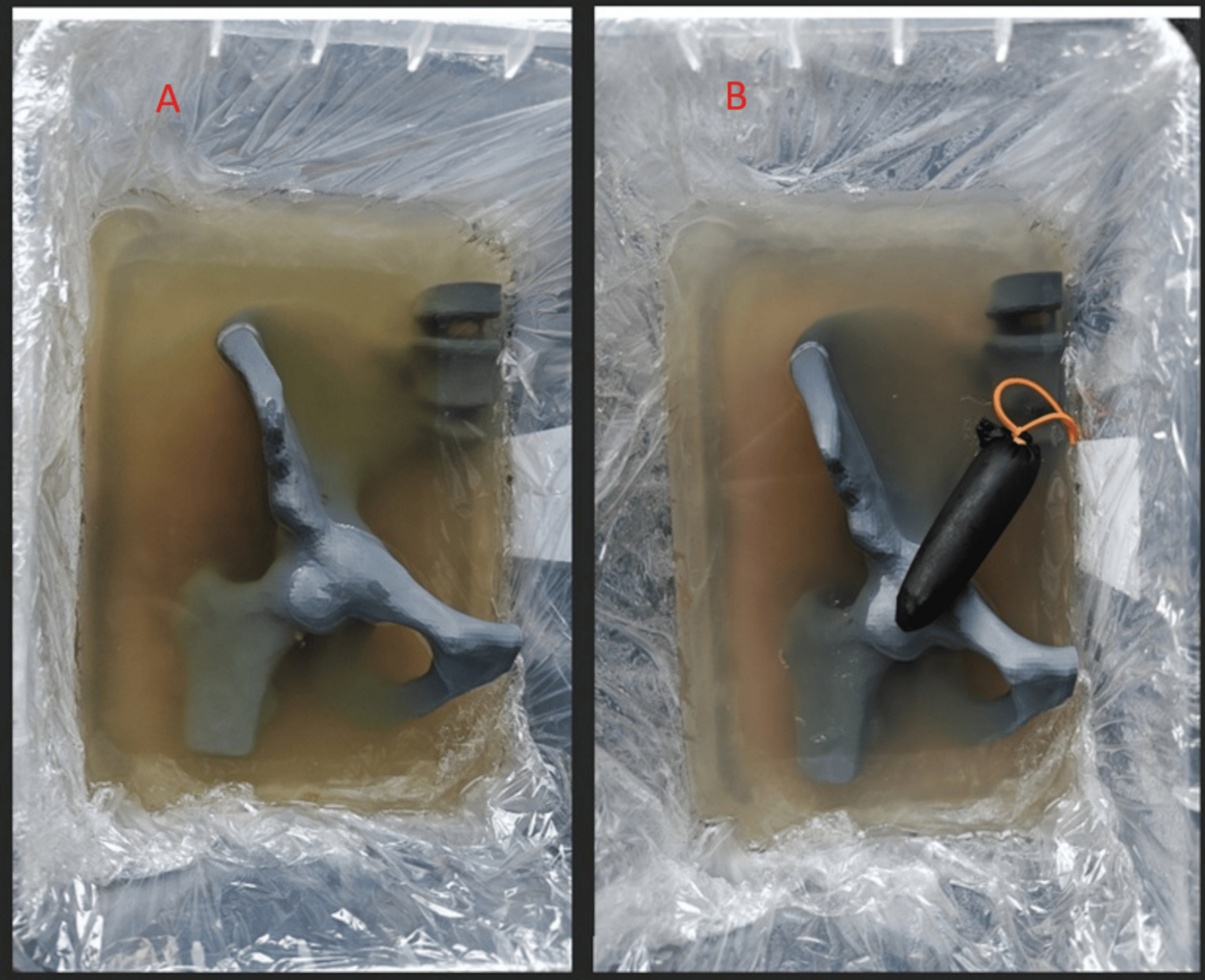
Post-initial gelatine pour (A) and with the replica psoas tendon in situ (B) (A) The initial gelatine pour to the level of the iliac brim. This was left to set for six hours in a refrigerator. (B) The replica psoas tendon, comprising spaghetti strands contained within the finger of a nitrile glove, is positioned over the pelvic brim in the expected anatomical location. A second and final gelatine pour followed, completing the phantom once cured.

Specifically, 54 g of gelatine (Dr. Oetker Gelatine Sachets - SuperValu; Dr. Oetker, Bielefeld, North Rhine-Westphalia, Germany) was dissolved in 1.5 L of hot (not boiling) water and poured over the model to the level of the iliac brim (Figure 2A). This was left to cure in a fridge at 4°C for six hours. Post setting, a replica psoas tendon (see paragraph below) was fixed in place over the expected anatomical position using double-sided tape, prior to repeating the pour (54 g gelatine in 1.5 L of warm water) to completely submerge the 3D print. Utilising 3 L of gelatine in this container, this left roughly 3.5 cm of depth from the surface to the target site lateral to the psoas tendon. This was then left to set overnight in a fridge, again at 4°C.

This simple gelatine provides a basic medium for ultrasound scanning and the practice of the needling technique, but with relatively low soft-tissue fidelity. Additional measures are examined in the discussion section that may improve realism, if required.

Creation of the replica psoas tendon

Given the importance of the psoas tendon as a landmark in carrying out the PENG block, multiple attempts at achieving a sonographically realistic structure to represent it were made. While designed only for a sonographic target to be avoided, it does offer more resistance to needle advancement than the gelatine soft tissue if inadvertently punctured - alerting operators to needle misplacement.

Ultimately, acceptable results were obtained by inserting multiple strands of spaghetti pasta into the removed middle finger of a nitrile glove. Enough pasta was used to ensure the nitrile was stretched taut, and the length of the surrogate tendon was roughly 9 cm.

The structure was submerged in the gelatine mixture to allow de-aeration before being tied off. It was subsequently affixed to the pelvic brim of the 3D-printed model using double-sided tape (Figure 2B), before being totally submerged during the second gelatine pour.

When fixing the replica tendon, it is worth noting that, anatomically, the psoas tendon courses from the lumbar vertebral bodies, over the pelvic brim, to insert on the lesser trochanter of the femur. Aligning it in this direction on the 3D print places it naturally into the deepest part of the constructed ilium, where it would be anatomically expected to lie.

A slow and gentle pouring technique is advisable so as not to displace the orientation of the replica tendon. This also reduced the formation of air bubbles; the few that developed dissipated quickly.

Ultrasonographic results

**Figure 3:**
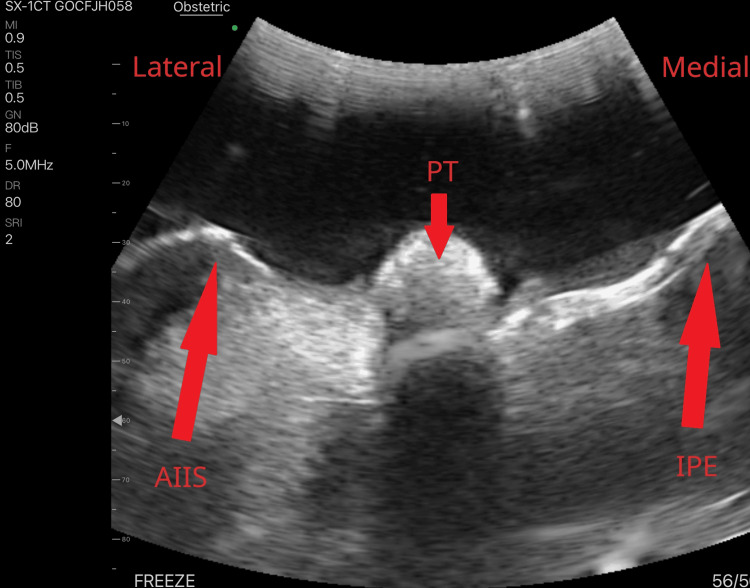
Ultrasound image of the constructed phantom AIIS = anterior inferior iliac spine; PT = psoas tendon; IPE = iliopubic eminence

Once fully cured, the phantom was assessed using handheld ultrasound (SonoHealth D5CL tethered to iPad Air 5th generation, utilising MicroVue software, Guangzhou SonoHealth Medical Technologies Co., Ltd., Guangzhou, China). A curvilinear probe was utilised at 5.0 mHz and 8 cm depth (the minimal depth feasible) in order to obtain a wide field of view, as is typical with performing a PENG block in real-life circumstances (Figure 3). The probe was orientated along the line of the pelvic brim, in a roughly 45-degree oblique orientation, with the anterior inferior iliac spine superolaterally, and the iliopubic eminence inferomedially.

**Figure 4:**
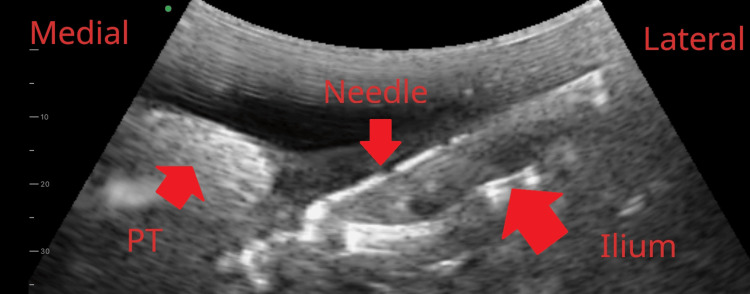
Needle insertion PT = psoas tendon

This orientation allowed visualisation of the 3D-printed replica of the pelvic brim and the psoas tendon surrogate. Soft tissue speckle was limited compared to real tissue. This orientation permitted needling attempts, as shown in Figure 4. 

Storage and durability

The durability of the model was reasonable, with virtually no surface damage to the gelatine layer despite probe scanning and compression. Needle insertion utilising an echogenic block needle (Ultraplex 360, 22G, 50 mm; B. Braun Melsungen AG, Melsungen, Germany) was feasible, enabling practice of the hand-eye coordination and block technique with advancement to the bony endpoint lateral to the psoas tendon. A total of 30 needling attempts were tested.

These multiple passes with the needle led to persistent needle track marks in the phantom that are a known issue with gel-based phantoms. These track marks became quite noticeable within five needle passes - but ultimately did not prohibit usage of the phantom for the stated purpose. Potential avenues to mitigate this artifacting are discussed in the discussion section.

The phantom was well preserved after initial testing by freezing at -10 degrees for over one week. Thawing of the model was achieved within four hours of being left at room temperature, and the phantom demonstrated no subjective deterioration in ultrasonographic fidelity after this thaw cycle.

However, within one day of being placed back in refrigeration at 4°C, the gelatine tissue substitute had begun to degrade with notable surface microbial growth. Further avenues for mitigation of this effect are discussed in the following section of this report.

Beyond this, shelf life was not tested across repeated use, freeze-thaw cycles, or longer storage periods.

Reusability

The 3D-printed component of the phantom is reusable. When the phantom has been used for its extended purpose, the print can be extracted from the gelatine and cleaned with soap and hot water, with the aid of a stiff-bristle brush, to remove any residual gelatine.

If an operator wishes to reuse the print in this fashion, it is necessary to close the resin drainage holes once the print is initially cured. This step prevents gelatine from entering the interior of the print, where it can be difficult to remove. This can be accomplished using any inexpensive adhesive putty, or Chitubox offers the option to retain the wall material removed during the hole creation process ( the "Keep the Hole" setting available in the "Dig Hole" menu). These offcasts can be printed and cured along with the main 3D print, and reinserted as plugs when required.

Cost

Regarding cost efficacy, the overall consumable materials cost of this phantom was 12.31 euros. The cost breakdown is summarised below (Table 2).

**Table 2:** Consumable costs

Component	Cost (euro)
Resin (Elegoo Space Grey - 184 mL)	5.33
Gelatine (Dr Oetker Sachets - 108 g)	6.87
Pasta (Supervalu Daily basics spaghetti - 50 g)	0.06
Nitrile glove (Single - Eiremed.ie)	0.05

This table does not include the cost of the 3D printer itself (279 euros at the time of writing), reusuable 99% isopropyl alcohol for washing, or curing equipment. While an inexpensive ultraviolet lamp was utilised in this instance (Navanino PZC-001 - 15 euros), technically prints can be cured in direct sunlight - but this typically takes longer and can be more labour intensive. Additionally, this table does not take into account the cost of PPE.

Electricity cost has likewise not been factored into the table due to variations in power draw associated with individual prints, but monochromatic SLA printers, such as those utilised here, are typically substantially more power efficient than fused deposition modeling (FDM) printers.

## Discussion

The present report supports construct feasibility and preliminary usability, but has not been formally validated for comparative fidelity or educational efficacy.

Feasibility

The overarching goal of this project was to harness the ability of home 3D printing technology to produce a viable phantom at a fraction of the cost of a more traditional, commercially available phantom. In addition, this was completed as simply as feasible - making it potentially attractive to replicate at short notice, given that it has minimal steps and a fast set-up time. As mentioned previously, this model has not yet been formally validated as a training tool. Even if a department were to consider the outright purchase of a home 3D printer, PPE, and curing equipment, the total cost would still amount to only a fraction of the cost of a single commercial phantom.

While the 3D printing (including pre- and postprocessing) does contribute to about nine hours of preparation time, embedding the finished print in the simple gelatine mixture could be achieved with a total of 12 hours of setting time. Given the reusable nature of the 3D-printed component, this would make subsequent construction of the phantom much faster.

Adding additional anatomical complexity

It must be acknowledged that, in the pursuit of creating a phantom that is as low-cost and simple as possible, some features have been foregone that could be considered if a department's needs dictated.

Firstly, the typical ultrasound image obtained for a PENG block will include other anatomical structures that have been omitted from this feasibility report - namely, the femoral artery, vein, and nerve. These lie superficially and medially to the psoas tendon, not in the path of needle advancement from supero-lateral to infero-medial, and given the added complexity of including these structures, they were omitted for expediency.

For those seeking a more comprehensive anatomical model, a variation of this project was constructed utilizing bundled rubber bands to represent the femoral nerve, and two further nitrile glove fingers filled with water to represent the great vessels (Figure 4). These were placed after submerging the replica psoas tendon under a layer of gelatine and allowing time for this second layer to cure. A third and final layer of gelatine was then poured to submerge the replica nerve, artery, and vein.

**Figure 5:**
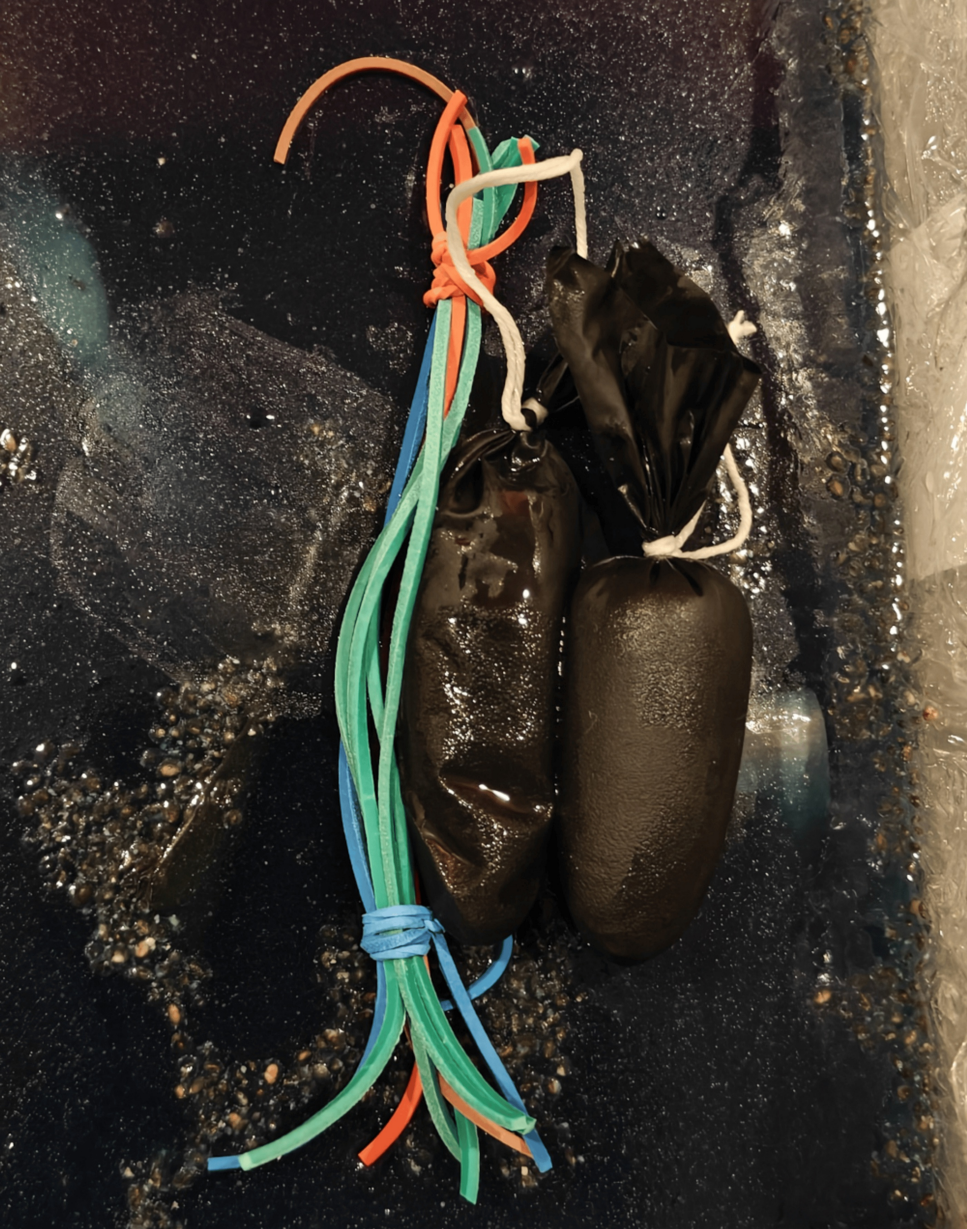
Addition of replica femoral nerve, artery, and vein components superficial and medial to the submerged psoas tendon

While providing a more realistic ultrasound image, this does entail an additional gelatine pour with associated setting time. Furthermore, any inconsistency in gelatine formulation may be noticeable as an obvious sonographic lamination layer on the ultrasound image. This is significantly more noticeable than any similar layer line occurring at the level of the pelvic brim between the first and second gelatine pours, as the 3D-replicated bone of the ilium effectively obfuscates this artifact.

Another potential modification that could be considered is the addition of a food dye to the gelatine mixture. This has the effect of obfuscating the 3D-printed pelvic brim and associated structures from casual observation, forcing ultrasound operators to put more effort into finding the optimal image on screen. Beyond a marginally increased cost, the downside is negligible. 

Improving ultrasonographic soft tissue fidelity

Utilising gelatine is a fast way to achieve a gel-based tissue-like material for the phantom; however, a potential disadvantage is that simple gelatine does not look particularly like muscular tissues under ultrasound imaging. Such tissue typically has a more speckled/grainy appearance on ultrasound that plain gelatine lacks. Multiple simple solutions to this issue have been devised for use in home-made ultrasound phantoms, such as incorporating metamucil/psyllium husk [17] and ground chia seeds [18]. This can improve the overall image quality and potentially make track marks from prior needle passes less noticeable. It does, however, exacerbate the potential lamination issue exhibited with multiple gelatine pours - with a degree of sedimentation evident at the bottom of each layer, which can lead to obvious and distracting ultrasonographic artifacts. For this reason, these techniques were omitted in this instance.

Avenues for improving shelf life

A known issue of gelatine-based phantoms is their short shelf life. This is typically limited unless frozen, owing to microbial contamination and surface growth. As discussed previously, freezing the phantom at -10 degrees was not associated with any detrimental effects to the phantom once adequately thawed, but within 24 hours or refrigeration after this thaw, the gelatine had begun to spoil.

Multiple potential additives have been considered to prolong said shelf life - with one source suggesting a potential approach being the addition of 10% povidone-iodine to the surface of the phantom [19]. Readily accessible surgical decontamination solutions such as 4% chlorhexadine (Hibiscrub) could also be considered, but were not formally evaluated in this feasibility study. 

Alternative 3D printing technologies and printing safety

Finally, the choice of using an SLA 3D printer rather than the more commonly used FDM printer technology might be of interest. FDM has gained popularity due to its ease of use and low cost. It involves extruding plastic-based polymers and gradually building the model from the build plate up.

SLA, in contrast, involves the use of a liquid resin that photopolymerises upon exposure to UV light. Its main benefit is in superior detail, which was not a particular consideration in this project. Downsides of SLA printing include the need for postprocessing with isopropyl alcohol to remove excess uncured resin, and additional ultraviolet curing before the print is deemed safe to handle. There are also concerns about the production of volatile organic compounds involved in the curing and printing processes, necessitating additional PPE and ventilation [20]. Best practice with any 3D printing technology, FDM or SLA, remains to wear PPE such as respirators and operate the machinery in a well-ventilated area.

Regardless of the technology chosen for a department, such home printers can typically be purchased for between 200 and 500 euros.

Where speed is necessary, however, SLA printers typically outstrip FDM printers. The same 3D print, prepared for FDM printing using PrusaSlicer, would be estimated to take an additional one hour and 20 minutes to complete. While the overall power consumption of both FDM and SLA printers is quite low, FDM printers typically require a larger power draw per hour owing to the necessity of keeping the build plate heated.

## Conclusions

This technical report describes the process of creating a simple and ultra-low-cost phantom trainer to facilitate safe practice of the ultrasound image acquisition and needling steps of the PENG block by regional anaesthesia enthusiasts. Formal evaluation of fidelity, durability, user experience, and educational effectiveness has not yet been completed.

When combined with some basic do-it-yourself (DIY) skills, 3D printing offers an affordable approach to providing such training aids. With some very basic knowledge of free software, 3D models can be modified to suit a department's specific needs as described here. Modern home 3D printing technology has become eminently affordable, with the 3D printer used for this project costing a fraction of the cost of a commercially available USGRA phantom.
